# Supplementary material for: Copy number variants in the sheep genome detected using multiple approaches
Source: BMC Genomics. 2016 Jun 8;17:441. doi: 10.1186/s12864-016-2754-7 (PMC4898393; doi:10.1186/s12864-016-2754-7)
Supplement: Additional file 3: — A description of the results of the cross platform (385 K CGH and SNP50 chip) verification of CNV regions [72, 73]. (PDF 8 kb) [file 12864_2016_2754_MOESM3_ESM.pdf]

Cross platform verification of autosomal CNVRs using 385K and SNP50 data

A small subset of animals assayed with the 2.1M CGH array were also used for data generation with either a lower density 385K CGH array (5 individuals) or the OvineSNP50 BeadChip (24 animals; Supplementary Table 1). This facilitated an examination of the proportion of CNVRs independently called across platforms.

Using the 2.1 M CGH array, for the five reference animals a total of 935 CNVRs (1,268 CNV calls) were identified that could be mapped to genome BTA_OARv.2 for comparison to Roche-NimbleGen 385K CGH array results. Of these, only 13 CNVRs (and 17 CNV calls) had a corresponding segment call in the 385K CGH array dataset (Table 2). The average length of verified CNVR was 387kb, much larger than the average of CNVRs that were not verified using the 385K CGH dataset (30kb). Possible explanations for the very low verification rate (1.4%) are provided below, but are likely to be caused in part by the differences in the probe density between the two CGH arrays. This prompted the reverse comparison, whereby 52 CNV segment calls made using the 385K CGH array (with absolute $\log_2$ ratio threshold of 0.25) were examined within the larger 2.1 M CGH array CNV calls. Only 29% (15) of these calls overlapped CNVRs from the 2.1M CGH array.

A separate comparison was performed against OvineSNP50 BeadChip data. A total of 2,847 CNVRs were observed in the 24 animals common to both platforms (2.1M CGH array and OvineSNP50 BeadChip), arising from 7,416 CNVs. Of these, just three CNV calls (two CNVRs) overlapped CNVs called by cnvPartition (Illumina Inc., USA) analysis of the SNP data (Table 2). CNVs predicted by the DNAcopy software [67] using Illumina Ovine SNP50 BeadChip data verified more CNVRs than cnvPartition, with 101 CNVs corresponding to 64 CNVRs verified by DNAcopy CNV calls (Table 2). The three calls verified with the cnvPartition dataset were not verified by DNAcopy.

Overall, CNVRs were difficult to verify between CGH arrays and with the OvineSNP50 BeadChip. Partly, this may be due to the fact that the CNVRs identified with the 2.1M CGH array had to be aligned to different genomes for comparison with the 385K CGH array and OvineSNP50 BeadChip. There is also evidence to suggest that SNPs on SNP chips are often biased away from CNV regions [20, 72, 73]. Also, SNPs that were included on the SNP array were identified using an earlier version of the sheep genome (OARv1) than was used to design probes for the 2.1M CGH array. Therefore, there may be fewer repetitive regions included in the OARv1 genome, which would add to the paucity of SNPs in CNV regions. The average spacing of SNP probes in the sheep genome is one probe every 60kb, which makes it difficult to detect small CNVs, even in regions where probes are spaced relatively consistently, let alone in regions that have fewer SNPs. In combination, these factors may explain the low cross platform verification rate observed with the Illumina OvineSNP50 BeadChip.
